# Geospatial codistribution of tuberculosis and diabetes mellitus in Indonesia

**DOI:** 10.1186/s40249-026-01432-x

**Published:** 2026-03-30

**Authors:** Indra Dwinata, Tsheten Tsheten, Ansariadi Ansariadi, Fasil Wagnew, Kefyalew Addis Alene, I. Nyoman Sutarsa, Paula Moraga, I. Wayan Gede Artawan Eka Putra, Matthew Kelly

**Affiliations:** 1 Department of Epidemiology, Faculty of Public Health, Hasanuddin University, Jln Perintis Kemerdekaan Km.10, Makassar, South Sulawesi 90242 Indonesia; 2 National Centre for Epidemiology and Population Health, College of Law, Governance and Policy, Australian National University, Canberra, Australia; 3 Population Health Program, QIMR Berghofer, Medical Research Institute, Brisbane, QLD Australia; 4 School of Population Health, Faculty of Health Sciences, Curtin University, Bentley, WA Australia; 5 School of Medicine and Psychology, College of Science and Medicine, Australian National University, Canberra, Australia; 6 School of Public Health, Faculty of Medicine, Udayana University, Bali, Indonesia; 7 Computer, Electrical and Mathematical Sciences and Engineering Division, King Abdullah University of Science and Technology, 23955-6900 Thuwal, Saudi Arabia; 8 Geospatial and Tuberculosis Research Team, The Kids Research Institute, Nedlands, Western Australia, Australia

**Keywords:** Tuberculosis, Diabetes mellitus, Geospatial analysis, Indonesia

## Abstract

**Background:**

Tuberculosis (TB) and diabetes mellitus (DM) co-morbidity is a growing public health challenge, particularly in Indonesia, where TB incidence remains high and DM prevalence is increasing. DM co-morbidity is known to increase the risk of TB incidence and have negative effects on TB treatment outcomes. This study aims to analyze the geographical co-distribution of TB and DM and their sociodemographic determinants in Indonesia, to help inform public health response and targeting of screening programs.

**Methods:**

Using data from the 2023 Indonesian Health Survey (SKI), a nationally representative, population-based survey, we applied a Bayesian geostatistical model to estimate disease prevalence and assess associations with key sociodemographic factors.

**Results:**

The predicted TB prevalence varied from 0.1% to 3.0%, highest in eastern Indonesia, particularly Papua, while DM prevalence ranged from 0.6% to 6.2%, concentrated in Java and Sumatra. Approximately 62 districts showed more than a 50% posterior probability that both TB and DM prevalences simultaneously exceed their respective national thresholds. The proportion of the poor population is significantly associated with higher TB prevalence (0.106; 95% CrI: 0.039, 0.174), while population density has a strong positive correlation with DM prevalence (0.198; 95% CrI: 0.156, 0.241). Proportion of the poor population (− 0.053; 95% CrI: − 0.096, − 0.009) and hospital services (− 0.071; 95% CrI: − 0.116, − 0.027) show a negative association with DM prevalence.

**Conclusion:**

Spatial analysis revealed significant regional variations, with high TB-DM co-distribution observed in rapidly urbanizing and high-poverty districts, including parts of West Java, East Java, Sumatra, and Kalimantan in Indonesia. These findings emphasize the need for strengthened TB-DM integration in healthcare services, especially in areas that have a high prevalence of both diseases. Strengthening integrated disease management strategies in local areas can help mitigate the burden of both TB and DM in Indonesia, particularly given likely low case detection and health care access in lower income regions.

**Graphical Abstract:**

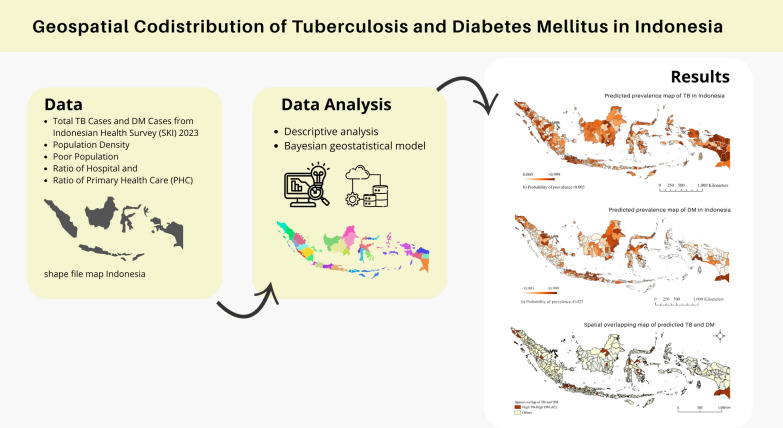

**Supplementary Information:**

The online version contains supplementary material available at 10.1186/s40249-026-01432-x.

## Background

The co-occurrence of tuberculosis (TB) and diabetes mellitus (DM) has become a major public health concern worldwide, impacting numerous countries. The incidence of TB is 2–5 times higher for those with diabetes compared with non-diabetics [1]. DM negatively impacts TB treatment outcomes, contributing to the progression of TB disease. Treatment outcomes are significantly worse for those with DM, and mortality risk is up to 6 times higher [2–4]. The global prevalence of TB patients experiencing DM comorbidity has been estimated at 13.73% [4].

Two-thirds of the total TB cases globally come from 8 countries, and Indonesia has the world’s second-largest TB burden [5]. The incidence rate of TB in Indonesia was estimated at 354 per 100,000 population and the mortality rate at 52 per 100,000 population in 2022 [5]. Indonesia has also experienced significant demographic and epidemiological transitions, resulting in a rising prevalence of DM [6]. Rapid urbanisation, population ageing, and lifestyle changes linked to economic development have driven a surge in non-communicable diseases, particularly DM [7–9]. The age-adjusted prevalence of DM has increased from 5% to nearly 10% among adults during the past decade in Indonesia [10]. Significantly, almost fifty per cent of diabetics in Indonesia remain undiagnosed or are not having their condition managed [11]. In low- and middle-income countries, such as Indonesia, where TB incidence is elevated and DM prevalence is rising [12], the comorbidity of TB and DM may hinder the attainment of global End TB goals to reduce TB incidence by 90% and TB mortality by 95% from 2015 to 2035 [13]. In Indonesia, DM is the third largest cause of TB incidence, following smoking and undernutrition [14].

Following the WHO Collaborative Framework for Care and Control of TB and DM, the Indonesian Ministry of Health is implementing bi-directional TB-DM screening programs to improve case detection and management [15, 16]. At present this program is being introduced in selected districts across Indonesia and is concentrating first on screening diabetic patients at primary healthcare centres for tuberculosis [17]. However, the completeness of this screening program has not yet been assessed. Initial results however indicate DM prevalence to be around 14% among TB patients. To optimise the targeting and implementation of these programs, understanding spatial disease distribution can play a critical role, particularly in a geographically diverse country like Indonesia [18, 19]. The geographical distribution of TB and DM can vary significantly across regions due to shared factors, including socio-economic conditions, environmental influences, and access to healthcare [20, 21]. In Indonesia, a clear divide exists between the distribution of DM and TB. Higher DM rates are observed in urban areas, driven by lifestyle and demographic shifts, while TB is concentrated in poor and overcrowded locales. This pattern demonstrates why spatial analysis is crucial for identifying where the concurrent burden is most intense and urgent [18, 22, 23]. Traditional epidemiological approaches often overlook these geographic patterns, limiting the ability to identify high-risk areas and allocate resources efficiently [21, 24]. By employing spatial analysis, we can map disease prevalence, detect clusters of higher disease burden, and identify spatial disparities that could inform targeted interventions [25–27]. However, the use of these methods for TB disease in Indonesia is so far restricted to the island of Java and small area, and spatial assessments of TB-DM co-morbidity have not yet been conducted [28–30]. While individual-level co-morbidity studies exist, limited research examines their spatial co-distribution across lower administrative levels in Indonesia using nationally representative data. Identifying high-risk areas for TB-DM co-morbidity is crucial for cost-effective public health interventions. This study aims to examine the spatial co-distribution of TB-DM and socio-demographic factors in Indonesia and to identify potential co-morbidity hot spots using spatial analysis and epidemiological modelling.

## Methods

### Study setting

Indonesia is the world’s largest archipelagic nation, consisting of more than 17,000 islands, with five main islands: Java, Sumatra, Kalimantan, Sulawesi, and Papua. The country spans a vast tropical region with varied climatic and topographical conditions, influencing population distribution and disease patterns [31]. Indonesia has a population of approximately 277 million people, making it the fourth most populous country globally [32]. The population is unevenly distributed, with Java hosting around 56% of the total population despite constituting only 7% of the country's land area [33]. Indonesia's government structure is decentralized, with a hierarchical administrative system that consists of four main levels: province, district, sub-district, and village. Indonesia's healthcare system comprises a mix of public and private providers, with the government running a national health insurance scheme known as Jaminan Kesehatan Nasional (JKN). Indonesia's healthcare system is categorized into three service levels, which are Primary Healthcare centers (Puskesmas, clinic or family doctors), Secondary referral healthcare facilities (general hospitals), and Tertiary healthcare facilities (advanced hospitals with highly specialised services) [33]. Services for the diagnosis, treatment and bidirectional Screening of TB-DM are available at all levels [34].

### Study design

An ecological study was conducted to map and predict the spatial distribution of TB and DM prevalence, estimate the relationship between socio-demographic factors and TB and DM prevalence, and identify districts with high prevalence of both TB and DM in Indonesia. The unit of analysis was the 514 districts in Indonesia.

### Data sources

Data on TB and DM cases was obtained from the aggregate data of the 2023 Indonesian Health Survey (SKI). The dataset is not publicly available, access requires formal application and approval from the Ministry of Health. SKI is a national survey with a cross-sectional design which was used to generate prevalence estimates of TB and DM at the district and national levels. Using probability proportional to size (PPS) sampling, the sample size was estimated at 345,000 households in 34,500 census blocks [35]. Data for the prevalence of TB and DM were weighted using sampling weight to ensure an equal probability of selection in the SKI 2023 data. A detailed explanation of the sampling techniques for the SKI can be found in another source [36].

Data on covariates related to TB and DM at the district level, including population density, poverty, hospital and health centre service ratio, were obtained from the Badan Pusat Statistik Indonesia (BPS) [32], Ministry of Home Affairs,[37] and Ministry of Health E-database, [38] BPS data are freely accessible through the official BPS website (https://www.bps.go.id), whereas data from the Ministry of Home Affairs and the Ministry of Health are not publicly available and require formal access approval.

### Variables and operational definitions

#### Outcome

Our primary outcome measures were the prevalence of TB and DM. We also generated estimates of the geographical overlap of TB and DM based on the predicted prevalence of TB and DM.

#### Covariates

The covariates used in this study have been identified to be associated with either TB, DM or both in other studies. These included population density, poverty prevalence, and hospital and health center service availability ratio [20, 39, 40].

#### Operational definition

See Table 1

**Table 1:** Variables and their operational definition as used in the study

Variable	Definition	Data source
Tuberculosis cases	Respondents who had been diagnosed with tuberculosis, in the year preceding the survey, by health workers through sputum examination, chest X-ray or both at all ages ≥ 1 year [35, 41]	Indonesian Health Survey 2023 https://www.badankebijakan.kemkes.go.id/data-mikro-ski/
Diabetes mellitus cases	Respondents diagnosed with diabetes mellitus by health workers in the population age group ≥ 18 years [35, 39]	Indonesian Health Survey 2023 https://www.badankebijakan.kemkes.go.id/data-mikro-ski/
Population density	Total population divided by the size of the area [42, 43]	E-database Ministry of Home Affairs Indonesia https://e-database.kemendagri.go.id/
Poor population	The number of people below the poverty line divided by the total population at the same period expressed in per cent [20]	Statistics Indonesia https://www.bps.go.id
Ratio of hospital	The ratio of the population to the number of hospitals in each district [44]	E-database Ministry of Health https://sirs.kemkes.go.id/
Ratio of primary health care	The ratio of the population to the number of primary health cares in each district [44]	E-database Ministry of Health https://www.kemkes.go.id/

### Data analysis

#### Descriptive analysis

This study utilized data from the 2023 Indonesian Health Survey (Survei Kesehatan Indonesia, SKI). The detailed methodology of the SKI is published elsewhere [36]. Prevalence of TB was calculated for each district among 877,531 survey participants in all age groups. For DM prevalence, the population analyzed was those aged above 18 years or 651,159. This cut-off was applied in accordance with the SKI 2023 survey protocol, which defines diabetes indicators only for adults. Restricting the analysis to individuals aged ≥ 18 years also ensures comparability with other national and international epidemiological studies on DM [39, 45].

### Geospatial model

We employed Bayesian model-based geostatistics (MBG) to investigate the spatial risk and identify socio-demographic and health system risk factors of TB and DM in Indonesia. Predicted prevalence models were constructed using binomial logistic regression models, by incorporating fixed and random effects. The correlation between variables was assessed using Pearson’s correlation coefficient (supplementary file (S1). Variance inflation factor (VIF) was also calculated using linear model to test the multicollinearity issues. All VIFs were below 0.5, indicating no serious multicollinearity concerns (S2).

During the preparation of spatial data for modelling, invalid geometries within the Indonesia shapefile was encountered, specifically involving duplicate vertices shared between polygon edges. These errors indicated topological inconsistencies within polygon geometries. To resolve this, we used *st_make_valid* function in *sf* package, which reconstructs invalid geometries into valid forms by correcting structural flaws. Cleaning this data was crucial because invalid geometries can break neighbor detection and distort the spatial weight matrix. All district geometries were validated prior to constructing the spatial adjacency structure. With all geometries valid, *poly2nb* function from the *spdep* package in R was used to identify neighbors using distance-based approach in the main analysis. Regions with no neighbors—typically those isolated polygons—were retained in the analysis by enabling zero.policy = TRUE during matrix construction, allowing them to be included with zero-weighted rows. The resulting adjacency list was subsequently converted into a graph format compatible with the INLA framework for spatial modelling.

We developed six different models for each disease (DM and TB), however, only the result of the final model is presented here (Table 3), with details presented as a supplementary file (S3). Below, we demonstrate the model development process for DM. Similar approaches were followed for TB.

### Model specification

We modeled the prevalence of TB and DM using a Bayesian geostatistical framework. For each district, ${Y}_{j}$ denote the number of TB and DM cases and ${N}_{j}$ the number of people surveyed at location $j$. Let ${p}_{j}$ denote the modelled prevalence at location $j$. Then ${Y}_{j}$ is modelled using a binomial distribution (likelihood) as:

$${Y}_{j} \sim Binomial ({N}_{j}, {p}_{j})$$

where:

${Y}_{j}$ is the number of observed cases,

${N}_{i}$ the number of individuals surveyed, and

${p}_{j}$ the modelled prevalence at district $j$

Using logit link function (which transforms the probabilities in [0, 1] interval to real values in ($-\infty , +\infty )$, the association between predicted prevalence and linear combination of predictors is expressed as:

$$\mathrm{logit}\left({p}_{j}\right)=\mathrm{log}\left(\frac{{p}_{j}}{1-{p}_{j}}\right)=\alpha +\sum_{k=1}^{k}{\beta }_{k}{X}_{jk}+{u}_{j}$$

where:

- ${p}_{j}$ is the probability of diabetes in area *j*.
- $\alpha$ is an intercept.
- $\sum_{k=1}^{k}{\beta }_{k}{X}_{jk}$ is a linear combination of predictors, where:
- ${\beta }_{1}$ (effect of population density).
- ${\beta }_{2}$ (effect of poverty).
- ${\beta }_{3}$ (effect of Hospital service availability).
- $${\beta }_{4} (\text{effect of PHC service availability})$$
- ${X}_{jk}$ is the value of covariate *k* in the area *j*
- ${u}_{j}$ is a spatial random effect capturing both structured and unstructured spatial variation.

As shown above, to account for the spatial autocorrelation at the district level, we used conditional autoregressive (CAR) prior distributions ${u}_{j}$[46, 47]. We implemented Besag-York-Mollié (BYM2) model, which accounts for both structured and unstructured random effects [48]. BYM2 model provides flexibility for modelling both spatial and local heterogeneity. We modelled ${u}_{j}$ as follows:

- $${u}_{j}$$ = $$\sqrt{\varnothing }$$*.*$${S}_{j}$$+$$\sqrt{1-\varnothing }$$*.*$${V}_{j}$$
- ${S}_{j}$ is the structured spatial effect (ICAR)
- ${V}_{j}$ is the unstructured effect (IID)
- $\varnothing$ is the mixing parameter (spatial structure weight) representing of total spatial variance attributed to the structured component

Spatial relationships between districts were structured using an adjacency matrix $W$, with elements ${w}_{ij}$ composed of zeros and ones, where ${w}_{ij}=1$ if and only if districts $i$ and $j$ are neighbors ($i\sim j)$, 0 otherwise.

$${w}_{ij}=\left\{\begin{array}{c}1, ifdistricts i and j are neighbors\\ 0, otherwise\end{array}\right.$$

Then, the CAR model is represented by the following conditional distribution:

$${S}_{j}|{S}_{-j}\sim N\left(\frac{1}{{w}_{j+}}\sum_{i\sim j}{S}_{i}, \frac{1}{{\tau w}_{j+}}\right)$$

where, $i\sim j$ indicates that district $i$ is a neighbour of district $j$

${w}_{j+}$ represents the “number of neighbors” for district$j$,, and

$\tau$ is the precision parameter (inverse of variance)

All analyses were performed in R version 4.5.1 (R Core Team, Vienna, Austria). We used the Integrated Nested Laplace approximation (INLA) method to approximate Bayesian inference [49]. For prior specification, we selected default vague priors for both parameters and hyperparameters implemented in R-INLA package. The posterior mean and 95% credible intervals (CrIs) were estimated for all the parameters. Along with spatial predicted prevalence maps, we also calculated exceedance probabilities for both TB and DM using thresholds that would be useful for policymaking. In both cases, the national prevalence was used as a threshold for each disease. For each district, we calculated the proportion of posterior samples where both predicted prevalences exceeded 0.025 (2.5%) for DM and 0.0028 (0.28%) for TB. This approach accounts for joint uncertainty and spatial correlation, enabling probabilistic identification of hotspots.

Furthermore, we created overlapping maps for DM and TB to explore spatial patterns. Districts that have prevalence values in quintiles 4 and 5 were categorised as high prevalence, while those in quintiles 1 and 2 were categorised as low prevalence. This approach allows for the detection of regions where the prevalence of two or more diseases reaches its highest point simultaneously.

Deviance information criteria (DIC) and Watanabe-Akaike Information Criterion (WAIC), were used for investigating the goodness-of-fit and selecting the most parsimonious model supplementary file (S4) [50]. In addition, we also employed root mean square error (RMSE) and Probability Integral Transform (PIT) technique to evaluate the model performance. We performed fivefold cross-validation to calculate RMSE evaluate the predictive performance of the model. Each fold served as a test set once, with predictions generated for held-out districts. The model incorporated spatial structure and accounted for population size. We also calculated PIT values, to evaluate the performance of the model. For each district, PIT values were calculated using the cumulative distribution function of the normal approximation to the binomial, based on the observed proportion of TB and DM cases and the model-predicted mean and variance. A histogram of these PIT values was then plotted to visually inspect model fit [49, 51].

For sensitivity analysis, a distance-based approach was employed to define neighborhood relationships, using the *dnearneigh* function from the *spdep* package in R. District centroids located within a 100 km threshold were considered neighbors, capturing epidemiologically meaningful proximity across Indonesia’s geographically fragmented island system [52, 53]. This approach allows for spatial correlation between districts separated by short sea distances while avoiding artificial disconnection of island regions. Additionally, we explored a weighted regression model using survey sampling weights. However, several districts had weights equal to zero, which caused computational instability and prevented successful model execution. To address this, we assigned negligible weights (0.001) to those districts for testing purposes. The results remained consistent with the unweighted model and were presented as a supplementary file 5.

## Results

### Prevalence of TB and DM by sociodemographic characteristics

According to the 2023 Indonesian Health Survey (SKI), TB prevalence stands at 0.30%, while DM was considerably higher at 2.37%. Prevalence of both diseases increased with age, with significantly higher rates observed among those aged 40 years and above, TB at 0.42% and DM at 4.37%. Gender differences were evident, with males having a higher TB prevalence (0.38%) compared to women (0.22%), while women showed a greater burden of DM (2.85%) than men (1.90%), with these differences being statistically significant in each case.

Urban residence, lower education levels, and informal employment were consistently associated with higher prevalence for both TB and DM. TB prevalence was slightly higher in urban areas (0.32%) compared to rural areas (0.27%), with individuals having lower education (0.33%) and informal employment (0.40%) being affected more. A similar pattern was seen for DM, with urban residents (2.90%), less educated individuals (2.62%), and non-working individuals (2.90%) showing the highest prevalence. All these differences were statistically significant (Table 2).

**Table 2:** Weighted prevalence of tuberculosis (TB) and diabetes mellitus (DM) by sociodemographic characteristics Indonesia, 2023

Variable	Total population (*n*)	Weighted prevalence of TB (%, 95% *CI*)	*P*-value	Total population (*n*)	Weighted prevalence of DM (%, 95% *CI*)	*P*-value
Total	877,531	0.30 (0.28–0.32)		602,982	2.37 (2.31–2.44)	
Age groups (year)						
< 18	274,549	0.22 (0.19–0.26)	< 0.001	-		< 0.001
18 − 29	112,273	0.22 (0.17–0.27)		112,273	0.09 (0.07–0.13)	
30 − 39	136,717	0.27 (0.22–0.33)		136,717	0.46 (0.41–0.53)	
40 +	353,992	0.42 (0.38–0.46)		353,992	4.37 (4.25–4.49)	
Gender						
Male	414,262	0.38 (0.35–0.42)	< 0.001	273,590	1.90 (1.82–1.98)	< 0.001
Female	463,269	0.22 (0.19–0.24)		329,392	2.85 (2.76–2.95)	
Place of residence						
Urban	469,549	0.32 (0.29–0.35)	0.024	323,091	2.90 (2.81–2.99)	< 0.001
Rural	407,982	0.27 (0.24–0.30)		279,891	1.63 (1.55–1.71)	
Education						
Low (≤ junior high school)	521,375	0.33 (0.30–0.36)	< 0.001	334,564	2.62 (2.53–2.71)	< 0.001
High (≥ senior high school)	269,810	0.23 (0.20–0.27)		268,418	2.07 (1.99–2.16)	
Occupation						
Not work/school	311,243	0.26 (0.23–0.30)	< 0.001	204,554	2.90 (2.78–3.01)	< 0.001
Formal employment	171,576	0.28 (0.23–0.32)		170,960	2.26 (2.16–2.37)	
Informal employment	175,723	0.40 (0.36–0.46)		175,113	1.62 (1.52–1.71)	

Counts (*n*) are number of individuals or cases observed in the survey. Weighted prevalence (%) was estimated using survey sampling weights to account for the complex survey design and ensure national representativeness. *CI* Confidence interval

### Spatial association between TB, DM and covariates

From the comparison of logistic regression models for explaining spatial heterogeneity, we found the full BYM2 model with covariates (model 6) had the lowest DIC and WAIC values for both DM and TB and was used for the interpretation of the findings (supplementary file S4). The results in Table 3 illustrate that the proportion of the poor population (0.015; 95% CrI: 0.005, 0.024) is significantly and positively associated with TB. Population density (0.025; 95% CrI: − 0.008, 0.057) shows a positive association with TB, although the effect is not statistically significant, the same is true for PHC and hospital health services. For DM, population density (0.059; 95% CrI: 0.039, 0.080) has a significant positive association with DM. The proportion of the poor population (− 0.007; 95% CrI: − 0.013, − 0.001) shows a significant negative association with DM, and also hospital services − 0.001 (− 0.002, − 0.000).

**Table 3:** Regression coefficients for random and fixed effects of TB and DM

Variable	TB	DM
	Coeff, Posterior mean (95% Crl)	Coeff, Posterior mean (95% Crl)
Fixed effect		
Intercept	− 6.032 (− 6.102, − 5.964)	− 3.819 (− 3.862, − 3.776)
Population density	0.025 (− 0.008, 0.057)	0.059 (0.039, 0.080)
Poor population	0.015 (0.005, 0.024)	− 0.007 (− 0.013, − 0.001)
Hospital services	0.000 (0.000, 0.001)	− 0.001 (-0.002, − 0.000)
PHC services	− 0.003 (-0.006, 0.001)	0.002 (0.000, 0.004)
Random effect		
Precision	2.505 (2.031, 3.062)	5.011 (4.217, 5.907)
Proportion of structured effect	0.150 (0.042, 0.330)	0.029 (0.001, 0.126)

Population density expressed per 1000 persons/km^2^. Poor population measured as percentage of individuals below the poverty line. Hospital and PHC services measured as population-to-facility ratios (persons per hospital/PHC). *TB* Tuberculosis, *DM* Diabetes mellitus, *PHC* Primary health care, *PHC *Primary health care

Residual spatial variability was higher for TB than DM (since lower precision implies greater variance). Spatial Random Effect for TB (0.150; 95% CrI: 0.042, 0.330) exhibits significant spatial variation, highlighting geographical heterogeneity in TB risk, while DM (0.029; 95% CrI: 0.001, 0.126) also shows spatial variability, albeit weaker (Table 3).

### Geospatial distribution of TB and DM prevalence

**Fig. 1:**
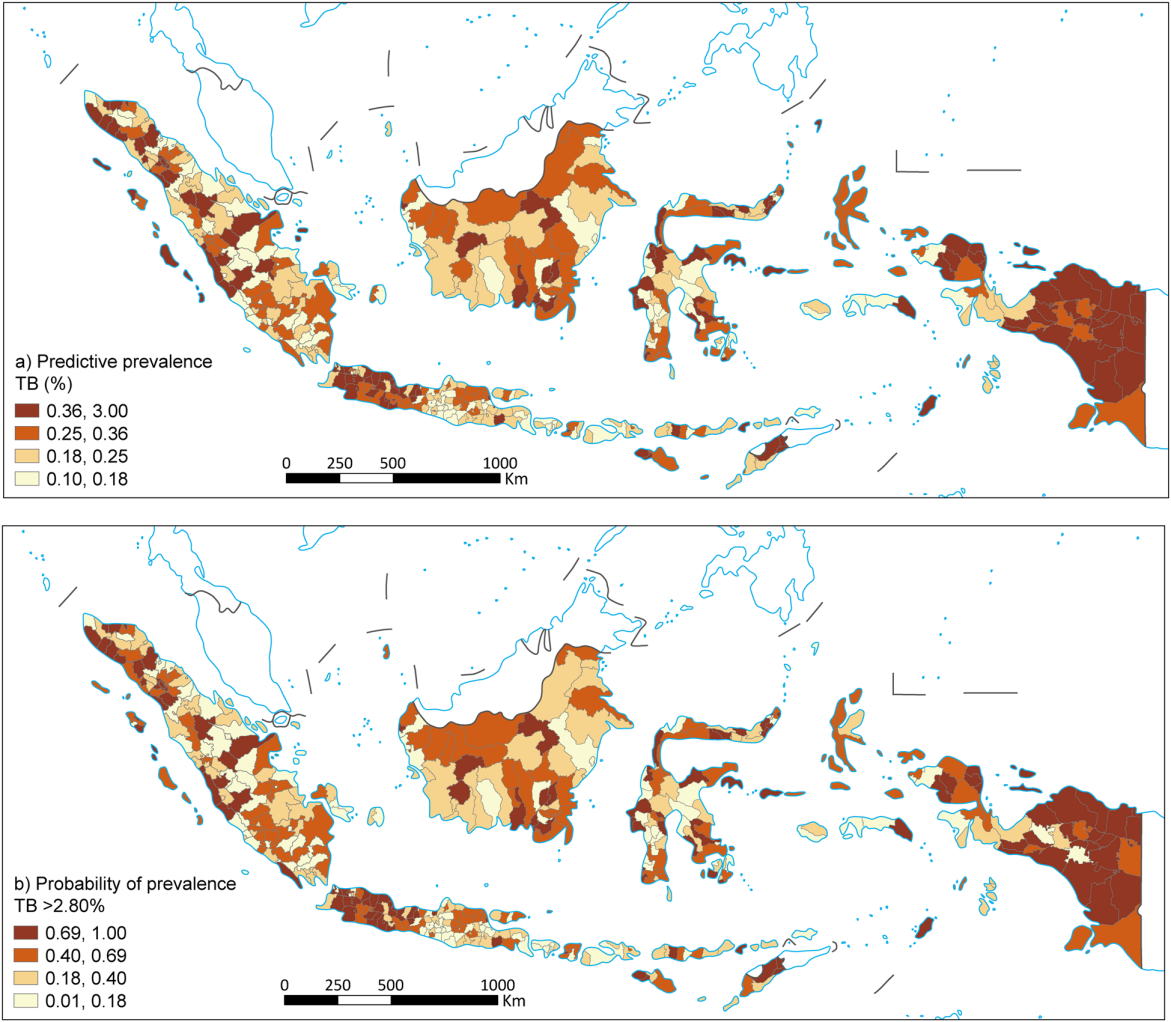
Predicted prevalence map of TB (**a**) and the probability that the prevalence TB (**b**). TB: Tuberculosis; DM: Diabetes Mellitus. Map approval number: GS(2026)0525

Figure 1a shows the spatial distribution of predicted TB prevalence across districts in Indonesia. The TB prevalence varies between 0.1% and 3.0%. Eastern Indonesia, particularly Papua, exhibits the highest TB prevalence. Several districts in West Java and Banten Provinces also had a high prevalence, in contrast, many parts of western Indonesia, such as Sumatra, East Java and Bali, showed lower TB prevalence. Figure 1b illustrates the probability that TB prevalence exceeds the national average of 2.80% in Indonesia. This map aligns closely with the predicted prevalence map, showing higher probabilities in regions where higher predicted prevalence was observed.

**Fig. 2:**
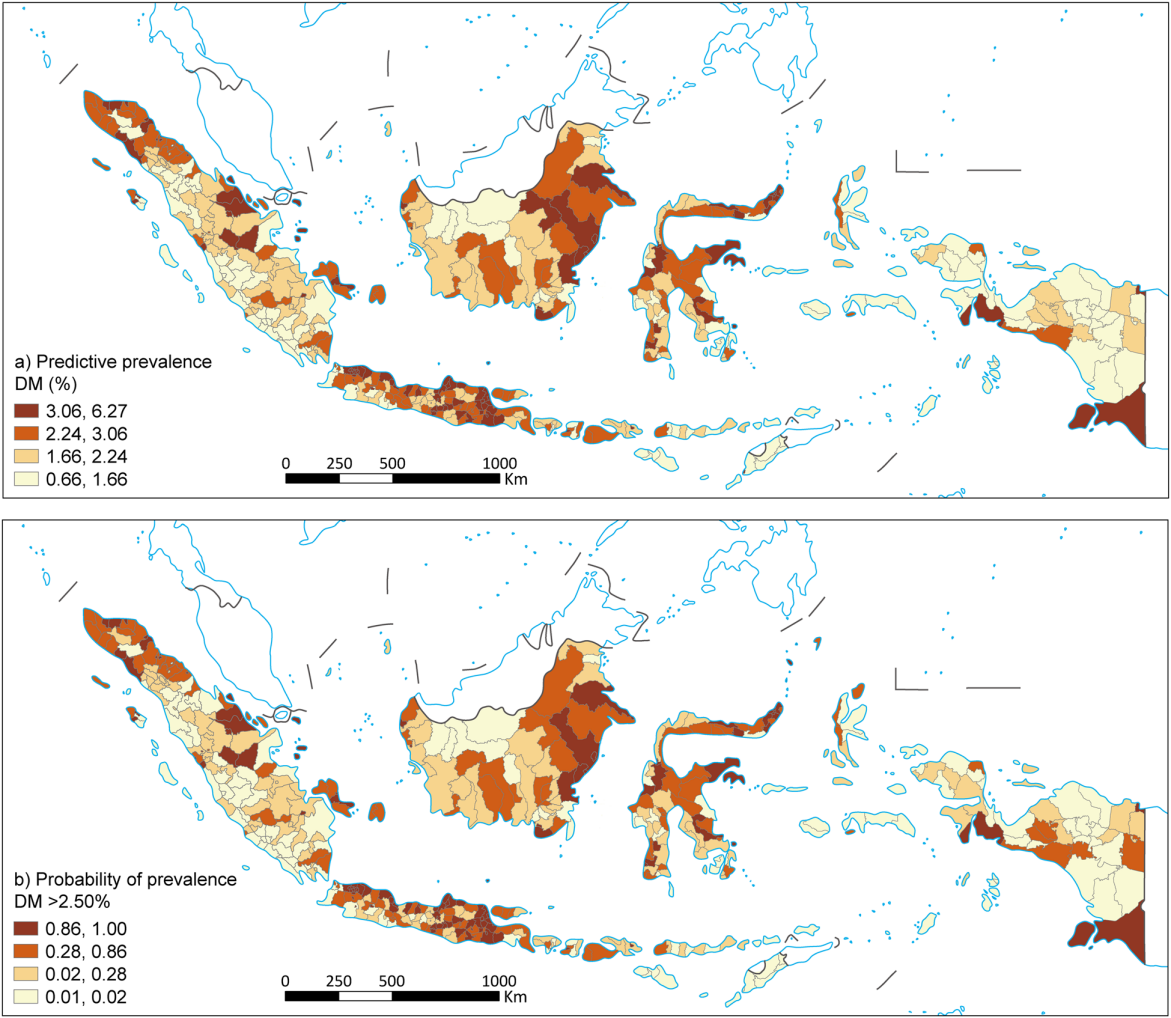
Predicted prevalence map of DM (**a**) and probability that the prevalence DM (**b**). TB: Tuberculosis; DM: Diabetes Mellitus. Map approval number: GS(2026)0525

Figure 2a shows the spatial distribution of predicted prevalence of DM across districts in Indonesia. The prevalence ranged from 0.66% to 6.27% with higher values predicted in West Indonesia in parts of Java and Sumatra (Central Java, East Java and Riau). The spatial distribution of DM prevalence is more heterogeneous than that of TB, reflecting a different pattern of disease risk. Likewise, the exceedance probability map showed higher probabilities in regions with a higher prevalence of DM (Fig. 2b).

**Fig. 3:**
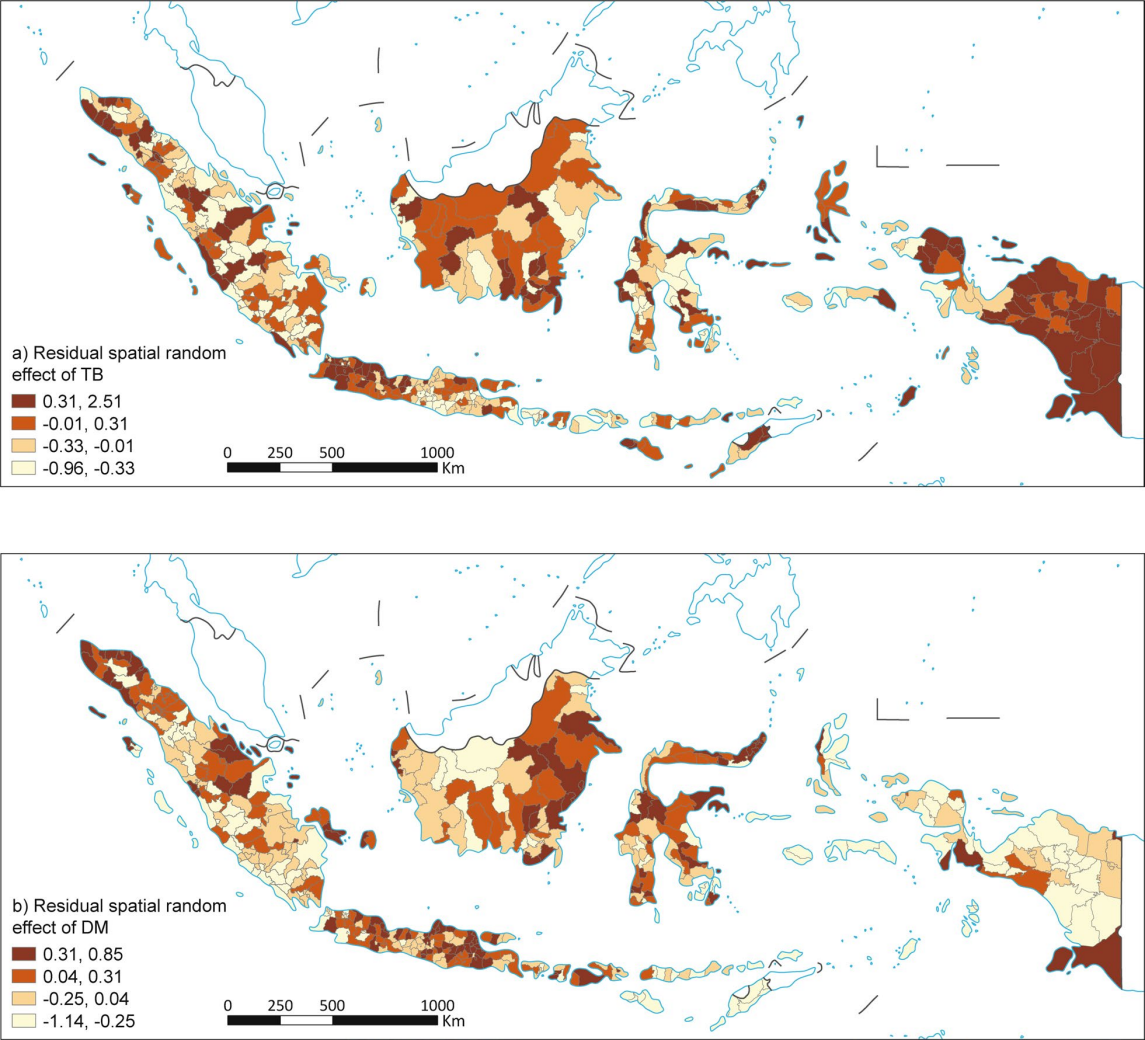
Residual spatial random effect of TB (**a**) and residual spatial random effect of DM (**b**). TB: Tuberculosis; DM: Diabetes Mellitus. Map approval number: GS(2026)0525

The map of the residual structured random effects (risk not explained by the model’s fixed effects) for TB showed notable spatial clustering across Indonesia. Darker regions indicating higher spatial clustering were observed in Aceh, Centre of Java, East Java, East Kalimanta, West Papua and North Sumatera provinces (Fig. 3a). DM on the other hand, presented a more widespread pattern of spatially structured unexplained risk. Provinces with higher spatial clustering included Central Papua, South Papua, Mountainous Papua, West Papua, South Kalimantan, DKI Jakarta and Bengkulu (Fig. 3b).

### Spatial overlap of TB and DM

**Fig. 4:**
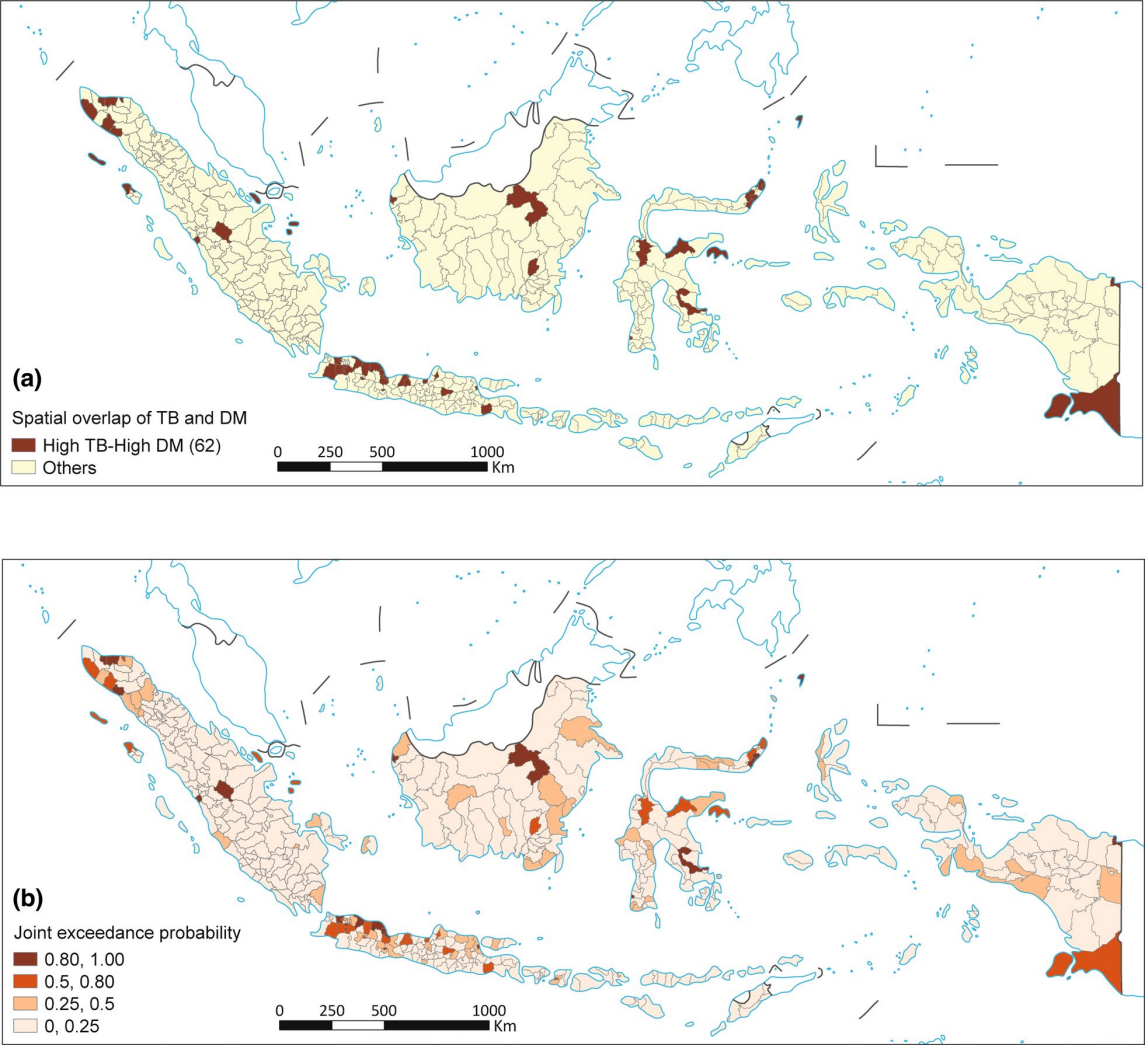
Spatial overlapping map of predicted TB (**a**) and DM (**b**). TB: Tuberculosis; DM: Diabetes Mellitus. Map approval number: GS(2026)0525

The joint exceedance probability map (Fig. 4) reveals distinct spatial patterns of co-occurring TB and DM risk across Indonesia. Approximately 62 districts demonstrated joint exceedance probabilities greater than 0.50, indicating that these areas have more than a 50% posterior probability that both TB and DM prevalences simultaneously exceed their respective national thresholds. A few districts exhibited a geographical overlap in the expected TB and DM prevalence, with the most significant clustering in West Java and Banten province, some areas in Aceh in the north of Sumatra Island, districts in Kalimantan Island (east Kalimantan and Center of Kalimantan), and some parts in the north of Sulawesi also had overlapping high TB and high DM.

## Discussion

This study highlights the significant spatial variation in both TB, DM and both diseases across Indonesia’s 514 districts. Noteworthy is the high levels of TB-DM co-distribution identified in urban, but high poverty, districts, particularly in the Western part of Java. These results indicate the need for strengthening integrated health services for TB-DM co-morbidity in these areas and the prioritisation of bi-directional screening programs.

### Spatial distribution of TB in Indonesia

This study showed spatial variation in TB prevalence among 514 districts in Indonesia. The geographical distribution of cases presented trends and proportions congruent with prior research and studies [19, 54], suggesting disparities related to health facilities, socioeconomic and environmental aspects in different regions. TB prevalence was concentrated in the Java region, including districts located in West Java, Banten and DKI Jakarta, as well as districts on Papua Island.

Our results also show prevalence of TB in Indonesia in 2023 is higher in urban areas, possibly due to higher housing density in cities than in villages, and the contribution of urban slums, increasing transmission risk. However, the Eastern Indonesian region of Papua has a high risk of TB despite the low population density in the region, which may be due to the high co-infection of TB with Human Immunodeficiency Virus (HIV) in the region [55]. High rates of malnutrition and stunting in Papua, may also contribute to a weakened immune system, making individuals more susceptible to TB [56, 57]. Papua is one of the most impoverished regions in Indonesia and access to healthcare is limited as many areas of Papua are geographically isolated [58].

### Spatial distribution of DM in Indonesia

The study also identified significant spatial variations in the relationship between socio-demographic variables and DM. Extreme diversity was observed in the prevalence of DM based on self-report. These results show that there is significant clustering and random distribution in the prevalence of DM in various regions, especially in Java Island, including East Java, DI Yogyakarta, and DKI Jakarta. This distribution pattern presents proportions that are in accordance with previous research [59]. The spatial pattern of DM prevalence is concentrated in high-population and urban areas. This may be due to higher economic development in urban areas which is often correlated with an increase in the burden of DM due to lifestyle changes. This is also in line with spatial research on DM globally, including in India, which found that there is a grouping of DM cases in the Southern part of India, which is a rapidly growing urban area [60]. While in Brazil the spatial pattern of DM individual socioeconomic characteristics and environment is influenced by individual socioeconomic characteristics and environment [61]. Importantly, urban areas have easier access to health services so they may report a higher prevalence of DM due to a higher likelihood of being screened and diagnosed.

Many provinces in the eastern part of the country, such as Papua, West Papua and Maluku report lower prevalence rates of DM in this study. However, this lower prevalence may not necessarily reflect a truly lower burden of disease, but Healthcare facilities in Eastern Indonesia are often more limited in terms of resources, workforce, and diagnostic equipment [62]. Primary healthcare centers (Puskesmas) in these regions may lack routine screening programs for DM, leading to underdiagnosis and underreporting. The potential underreporting and underdiagnosis bias for low-income areas has been noted in other studies of non-communicable diseases (NCD) prevalence in low and middle income countries (LMICs), whereas in higher income settings, DM is significantly more common in resource limited areas. Biased measurement has been observed to be particularly noteworthy in self-reported measures of NCD prevalence, as utilised in this study, when compared to symptom-based measurements [63].

### Spatial overlap TB and DM in Indonesia

The findings of this study highlight significant spatial heterogeneity in the prevalence of TB and DM across districts in Indonesia. Hotspots of TB and DM were identified in regions characterized by high population density and elevated urban poverty rates for instance districts in west Java, East Java, Aceh and several areas on the islands of Kalimantan, and Sulawesi, in congruence with similar studies identifying social determinants and local health system capacity as critical factors in the spatial distribution of these diseases [64–66]. These spatial disparities suggest that socio-demographic, socio-economic status, and healthcare-related factors, play a critical role in shaping the geographical distribution of TB and DM. This observation aligns with global trends where socioeconomically disadvantaged populations often experience a disproportionate burden of TB and DM, exacerbating health inequities.

In the Indonesian context, these findings are particularly relevant given the country’s dual burden of communicable and non-communicable diseases. Indonesia is the second-highest TB-burdened country globally. Meanwhile, the prevalence of DM among adults has nearly doubled over the past decade, and projections indicate a continued rise in DM prevalence in Indonesia, increasing the challenge for TB management [65]. TB-DM co-morbidity presents unique challenges to public health efforts, as DM increases the risk of developing active TB by two- to three-fold and worsens TB treatment outcomes. Additionally, the spatial clustering observed in this study mirrors findings from previous studies in Java, Indonesia, which linked high TB prevalence to factors such as poverty, poor and limited healthcare access [28]. This suggests that existing health inequities are driving the overlapping burden of TB and DM in specific districts, underscoring the need for localized interventions tailored to these vulnerable populations, for example, implementing Community-Based Integrated TB-DM Screening in High-Burden Urban Slums areas and Strengthening TB-DM Services in Remote Eastern Indonesia. These findings contribute to the growing body of evidence advocating for integrated and targeted approaches to TB and DM management in Indonesia. Policies addressing the shared risk factors for both diseases—such as improving access to healthcare services, reducing poverty, and promoting healthy lifestyles—are critical. For example, the implementation of bi-directional screening programs for TB and DM, as recommended by the World Health Organization (WHO), could enhance early detection and improve treatment outcomes. Beyond its policy implications, this study also provides important methodological contributions. The modeling framework enables national-scale, district-level mapping of TB and DM prevalence, allowing for spatially targeted planning. The use of exceedance probabilities identifies statistically defined hotspots where prevalence exceeds the national average, while the BYM2 spatial model disentangles structured regional clustering from unstructured local variation. Together, these approaches enhance the interpretive rigor and policy relevance of our findings. Addressing the spatial disparities through geographically targeted interventions could optimize resource allocation and reduce the health burden in high-risk districts. These findings highlight the need for a comprehensive and context-specific strategy to mitigate the impact of TB and DM co-morbidity on Indonesia's public health system while advancing global efforts toward achieving WHO End TB targets [67].

### Limitation of study

This study has several limitations that should be considered when interpreting the findings. First, the analysis relied on aggregated data from the Indonesian Health Survey (SKI) 2023, which may obscure individual-level variations in TB and DM co-morbidity. This ecological approach does not account for the individual risk factors and their direct interactions, potentially leading to ecological fallacy. Second, the study used secondary data sources, which may introduce inconsistencies due to variations in data collection methods and reporting accuracy across districts. In particular, the self-reported nature of the data relies on both good participant recall and the availability of screening services which would allow participants to be aware of their disease status. For instance, underreporting or screening programs for the detection of diseases in rural or resource-limited settings could have led to an underestimation of TB and DM prevalence in certain areas. This is particularly relevant in some parts of Eastern Indonesia included in our study, where DM prevalence was low but this could be due to this substantial under-diagnosis, and low health care access. The ecological study design utilised here also precludes the possibility of causal inference in our results. Finally, potential confounders, such as nutritional status and co-infections like HIV, were not included in the analysis despite their known impact on TB and DM prevalence. Addressing these limitations in future research, including the integration of longitudinal and individual-level data, could provide a more comprehensive understanding of TB and DM spatial in Indonesia.

## Conclusions

This study identified key patterns of spatial co-distribution in Indonesia, particularly in high population density, lower income urban areas in West Java/Banten, Aceh, East/Central Kalimantan, and parts of North Sulawesi. The results provide critical insights into the dual burden of TB and DM in Indonesia, highlighting the need for a geographically targeted approach to disease prevention and management that accounts for the diverse geographic and socio-economic conditions across regions. Within Indonesia’s decentralized healthcare systems, TB and DM control interventions must be implemented adaptively by local governments, considering the unique characteristics of each region. The integration of TB-DM services should be strengthened through community-based strategies, such as enhancing primary healthcare capacity, implementing co-located, bi-directional screening in local health centres (*puskesmas*), ensuring feasible and resourced referral pathways for both diseases and availability of treatment medications, and optimizing community health workers capacity to reach vulnerable populations. Additionally, resource allocation should prioritize high-burden areas, particularly impoverished urban settings and remote regions with limited healthcare access, taking particular note of likely under-diagnosis of DM in remote and low-income areas. A key approach will be to prioritise this resource allocation in top-decile districts identified for co-morbidity, and to develop integrated health information systems and screening and care monitoring procedures that will strengthen health systems and processes to achieve the government priorities of increasing screening completeness. Strengthening coordination between national and local governments in health program planning and financing will be key to ensuring effective interventions. Further studies incorporating spatiotemporal trends could support the development of evidence-based policies that enhance the sustainability and effectiveness of TB and DM control efforts in Indonesia.

## Supplementary Information

Additional file 1

Additional file 2

Additional file 3

## Data Availability

Data used in this study were obtained from the Indonesian government Ministry of Health Republik Indonesia under permission and we are unable to share these data further. Data access to the Indonesian National Health Survey 2023 can be applied for at this website: https://www.badankebijakan.kemkes.go.id/data-mikro-ski. The analysis code (R script) is available from the corresponding author upon reasonable request.

## Abbreviations

TB: Tuberculosis
*CI*: Confidence interval
DM: Diabetes mellitus
HIV: Human Immunodeficiency Virus
SKI: Survei Kesehatan Indonesia/Indonesian Health Survey
PHC: Primary health care
BYM2: Besag-York-Mollié Model
Crl: Credible interval
WHO: World Health Organization
NCD: Non communicable diseases
LMICs: Low and middle income countries
INLA: Integrated Nested Laplace Approximation
DIC: Deviance information criteria
WAIC: Watanabe-Akaike Information Criterion

## Declarations

### AI use statement

During the preparation of this work the author(s) used [Chat GPT, Grammarly, and Quillbot] in order to [improve language and readability of the paper]. After using this tool/service, the author(s) reviewed and edited the content as needed and take(s) full responsibility for the content of the publication.
