# Supplementary material for: Geospatial codistribution of tuberculosis and diabetes mellitus in Indonesia
Source: Infect Dis Poverty. 2026 Mar 30;15:37. doi: 10.1186/s40249-026-01432-x (PMC13034603; doi:10.1186/s40249-026-01432-x)

**Supplementary file (S1).** Pairwise correlation coefficient between variables included in the study.

| Variables | Population density | Poor population | Hospital services | PHC Services |
| --- | --- | --- | --- | --- |
| Population density | 1.00 | 0.01 | -0.23 | 0.64 |
| Poor population | 0.01 | 1.00 | 0.00 | -0.04 |
| Hospital services | -0.23 | 0.00 | 1.00 | 0.03 |
| PHC Services | 0.64 | -0.04 | 0.03 | 1.00 |
|  |  |  |  |  |

**Supplementary file (S2).** Variance inflation factor (VIF) of variables

| **Variables** | **VIF Value** |
| --- | --- |
| Population density | 1.887 |
| Poor population | 1.003 |
| Hospital services | 1.116 |
| PHC Services | 1.791 |

**Supplementary file (S3):** Spatial modelling specification

We developed Bayesian logistic regression models to investigate the spatial risk and identify covariates of the disease. Below are the six models we developed:

First, we fitted a model that captures the overall mean effect and unobserved heterogeneity in data by including only intercept and unstructured random effect. This model allows for individual variability across observations that is not explicitly explained by other predictors.

$\mathrm{logit}\left( p_{j} \right)=\log\left( \frac{p_{j}}{1-p_{j}} \right)=\alpha+u_{j}$

Second, we fitted a model that examines the association between DM cases and relevant covariates by including intercept along with population density, poverty rate, and hospital services as predictors. The covariates were scaled to ensure comparability. This model does not include any random effects or spatial components, focusing solely on the fixed effects of the predictors. This model allows us to assess the independent effects of these variables on DM, while accounting for the overall population size at risk.

$$\mathrm{logit}\left( p_{j} \right)=\log\left( \frac{p_{j}}{1-p_{j}} \right)=\alpha+\sum_{k=1}^{3} \beta_{k}X_{jk}+\emptyset_{j}$$

Third we fitted a spatial model to evaluate the effect of spatial structure on DM cases. This model includes an intercept and a spatial random effect using Besag model, which accounts for the spatial dependencies between neighbouring areas. The spatial component captures spatial variability accounting for the spatial autocorrelation. The spatial structure is defined by the adjacency matrix provided through the graph object (graph = Q).

$\mathrm{logit}\left( p_{j} \right)=\log\left( \frac{p_{j}}{1-p_{j}} \right)=\alpha+\emptyset_{j}$

Fourth, we fitted a spatial model to explore the relationship between DM and covariates while accounting for the spatial dependencies. The model includes intercept, covariates and a spatial random effect modelled using Besag structure.

$\mathrm{logit}\left( p_{j} \right)=\log\left( \frac{p_{j}}{1-p_{j}} \right)=\alpha+\sum_{k=1}^{3} \beta_{k}X_{jk}+\emptyset_{j}$

Fifth, we fitted a spatial model using the Besag-York-Mollié model to account for both structured and unstructured spatial variability in DM cases. The model includes an intercept and a combined structured and unstructured random effect. This model allows to account for the local spatial dependences and observed heterogeneity across the districts.

$\mathrm{logit}\left( p_{j} \right)=\log\left( \frac{p_{j}}{1-p_{j}} \right)=\alpha+\emptyset_{j}+u_{j}$

Finally, in model sixth, we fitted a Besag-York-Mollié model to assess the relationship between DM cases and covariates, while accounting for both spatial dependencies and unobserved heterogeneity. The model includes an intercept, covariates, and a structured random effect using the Besag structure and unstructured random effect to account for the variability not explained by the spatial structure.

$\mathrm{logit}\left( p_{j} \right)=\log\left( \frac{p_{j}}{1-p_{j}} \right)=\alpha+\sum_{k=1}^{3} \beta_{k}X_{jk}+\emptyset_{j}+u_{j}$

Explanation of the terms:

$\alpha$ is an intercept, $\sum_{k=1}^{3} \beta_{k}X_{jk}$ is a linear combination of predictors: $\beta_{1}$ (effect of population density); $\beta_{2}$ (effect of poverty); $\beta_{3}$ (effect of healthcare service availability); $\emptyset_{j}$is a spatial structured random effect, representing the spatial dependency, and $u_{j}$the spatially unstructured random effects capturing non-spatial variability.

**Supplementary file S4**. Table Comparison of logistic regression models for explaining spatial heterogeneity in TB and DM prevalence

| Model TB prevalance | Random Effects | Covariates | Description | DIC | WAIC |
| --- | --- | --- | --- | --- | --- |
| 1. IID Model | IID (unstructured random effect) | None | Captures unobserved heterogeneity | 2471.8 | 2457.5 |
| 2. Non-Spatial Model | None | Included | Captures only covariate effects | 3084.8 | 3074.6 |
| 3. Spatial CAR Model | Besag (CAR) spatial effect | None | Captures only spatial dependencies | 2502.5 | 2552.8 |
| 4. Full Spatial CAR Model | Besag (CAR) spatial effect | Included | Captures spatial dependencies and covariate effects | 2491.0 | 3074.6 |
| 5. BYM2 Model | BYM2 (structured + unstructured effects) | None | Accounts for both structured and unstructured random effects. | 2467.0 | 2451.8 |
| 6. Full BYM2 Model with Covariates | BYM2 (structured + unstructured effects) | Included | Captures spatial dependencies, unstructured variability, and covariate effects. | 2462.7 | 2446.9 |

| Model DM prevalance | Random Effects | Covariates | Description | DIC | WAIC |
| --- | --- | --- | --- | --- | --- |
| 1. IID Model | IID (unstructured random effect) | None | Captures unobserved heterogeneity | 3557.6 | 3520.0 |
| 2. Non-Spatial Model | None | Included | Captures only covariate effects | 5402.9 | 5431.9 |
| 3. Spatial CAR Model | Besag (CAR) spatial effect | None | Captures only spatial dependencies | 3657.5 | 3735.2 |
| 4. Full Spatial CAR Model | Besag (CAR) spatial effect | Included | Captures spatial dependencies and covariate effects | 3641.4 | 5431.9 |
| 5. BYM2 Model | BYM2 (structured + unstructured effects) | None | Accounts for both structured and unstructured random effects. | 3558.8 | 3521.9 |
| 6. Full BYM2 Model with Covariates | BYM2 (structured + unstructured effects) | Included | Captures spatial dependencies, unstructured variability, and covariate effects. | 3551.3 | 3519.5 |

**Supplementary file S4. PIT Histogram of TB and DM**

1. **TB**


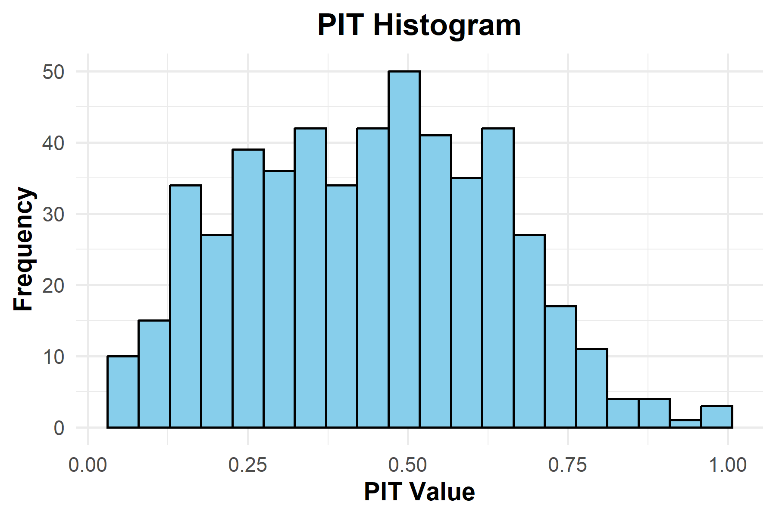


**b) DM**


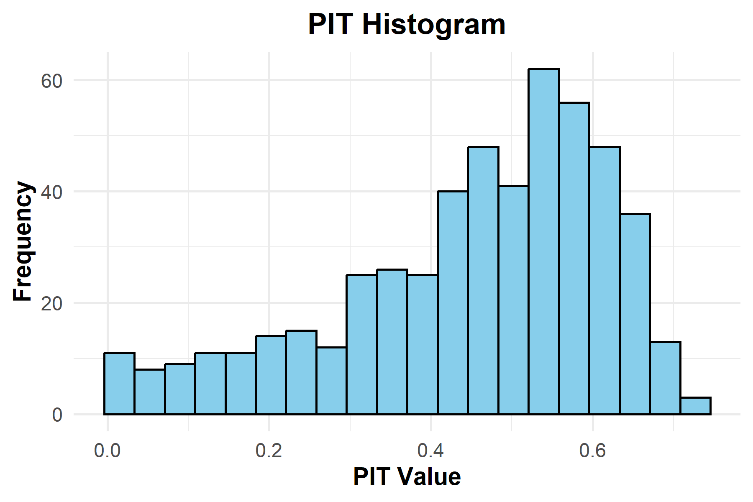

Supplement: Supplementary file 1 — Additional file 1 [file 40249_2026_1432_MOESM1_ESM.docx]
