# Supplementary material for: Geospatial codistribution of tuberculosis and diabetes mellitus in Indonesia
Source: Infect Dis Poverty. 2026 Mar 30;15:37. doi: 10.1186/s40249-026-01432-x (PMC13034603; doi:10.1186/s40249-026-01432-x)
Supplement: Supplementary file 3 — Additional file 3 [file 40249_2026_1432_MOESM3_ESM.docx]

**Supplementary file 6: R script for spatial modelling**

# Diabetes spatial modelling in Indonesia (Same steps involved for TB, only DM analysis is presented here)

# Steps:

# It includes importing the shapfile and cleaning the data

# Descriptive analysis included calculating summary statistics like mean and standard deviation

# Six models were constructed using queen contiguity matrix

# Joint exceedance was computed using the sixth model for both DM and TB

# Validation and sensitivity analyses conducted to validate and test the robustness of the model

# Load and Prepare Spatial Data

indonesia <- st_read("Map Indonesia (district level).shp")

# Check geometry validity

st_is_valid(indonesia)

# To know what the problem is

sf::st_is_valid(indonesia, reason = TRUE)

# Check if geometries are valid and fix invalid geometries

if(any(!st_is_valid(indonesia))) {

indonesia <- st_make_valid(indonesia)

}

# Checking if there is invalid geometries

sum(!st_is_valid(indonesia)) # should be 0

# Create the adjacency list using the Queen criterion

nb <- poly2nb(indonesia, queen = TRUE)

# Convert the adjacency list to an INLA graph format

Q <- inla.graph2matrix(nb2mat(nb, style = "W", zero.policy = TRUE))

# Import an merge covariate data with spatial data

cov_data <- read.csv("data.csv")

merged_data <- merge(indonesia, cov_data, by.x = "X_id", by.y = "id_map", all.x = TRUE)

# Renaming the variables

merged_data <- merged_data %>%

mutate(

pop_d = pop_density,

poor = percentage_poor_population,

hsp = rasio_HSP,

phc = ratio_PHC,

total_pop = resp_more18

)

# Convert the sf object to a data frame, dropping the geometry column

merged_data_clean <- merged_data %>%

st_drop_geometry()

# Ensure all variables are numeric for analysis

merged_data_clean <- merged_data_clean %>%

mutate(

pop_d = as.numeric(pop_d),

poor = as.numeric(poor),

hsp = as.numeric(hsp),

phc = as.numeric(phc)

)

# Create a summary table of the key variables

summary_table <- merged_data_clean %>%

summarise(

pop_d_mean = mean(pop_d, na.rm = TRUE),

pop_d_sd = sd(pop_d, na.rm = TRUE),

poor_mean = mean(poor, na.rm = TRUE),

poor_sd = sd(poor, na.rm = TRUE),

hsp_mean = mean(hsp, na.rm = TRUE),

hsp_sd = sd(hsp, na.rm = TRUE),

phc_mean = mean(phc, na.rm = TRUE),

phc_sd = sd(phc, na.rm = TRUE),

DM_more18_mean = mean(DM_more18, na.rm = TRUE),

DM_more18_sd = sd(DM_more18, na.rm = TRUE)

)

# Standardize all relevant covariates (pop_d, poor, hsp, phc)

merged_data_clean <- merged_data_clean %>%

mutate(

pop_d_scaled = scale(pop_d),

poor_scaled = scale(poor),

hsp_scaled = scale(hsp),

phc_scaled = scale(phc)

)

# Calculate the correlation matrix

cor_matrix <- cor(merged_data_clean %>% select(pop_d_scaled, poor_scaled, hsp_scaled, phc_scaled), use = "complete.obs")

# Check for multi-collinearity

model_tb <- lm(TB_cases ~ pop_d_scaled + poor_scaled + hsp_scaled + phc_scaled , data = merged_data_clean)

vif_tb <- data.frame(vif(model_tb))

# Model 1: IID model (only intercept and independent random effects)

merged_data_clean$re_u <- merged_data_clean$X_id # Assigning 'X_id' as the random effect variable

formula_iid <- DM_more18 ~ 1 + f(re_u, model = "iid")

result_iid <- inla(

formula_iid,

data = merged_data_clean,

family = "binomial",

Ntrials = merged_data_clean$total_pop,

control.predictor = list(compute = TRUE),

control.compute = list(dic = TRUE, waic = TRUE)

)

# Check the summary of the results

summary(result_iid)

fixed_effect <- result_iid$summary.fixed

precision <- result_iid$summary.hyperpar

fitted_values <- result_iid$summary.fitted.values

# model fit stat

model_fit <- data.frame(

DIC= result_iid$dic$dic,

WAIC = result_iid$waic$waic

)

model1 <- fixed_effect %>%

bind_rows(precision) %>% bind_rows(model_fit)

write.csv(model1, "model1.csv")

# Model 2) Non-Spatial Model with selected covariates (pop_d, poor, hsp)

formula_selected <- DM_more18 ~ 1+ pop_d_scaled + poor_scaled + hsp_scaled + phc_scaled

result_selected <- inla(

formula_selected,

data = merged_data_clean,

family = "binomial",

Ntrials = merged_data_clean$total_pop,

control.predictor = list(compute = TRUE),

control.compute = list(dic = TRUE, waic = TRUE)

)

summary(result_selected)

# Check the summary of the results

summary(result_selected)

fixed_effect <- result_selected$summary.fixed

fixed_effect1 <- fixed_effect %>%

rownames_to_column(var = "variable") %>%

rename(lci = '0.025quant',

uci = '0.975quant') %>%

mutate(mean = if_else(variable == "pop_d_scaled", mean/summary_table$pop_d_sd, mean),

sd = if_else(variable == "pop_d_scaled", sd/summary_table$pop_d_sd, sd),

lci = if_else(variable == "pop_d_scaled", lci/summary_table$pop_d_sd, lci),

uci = if_else(variable == "pop_d_scaled", uci/summary_table$pop_d_sd, uci),

mode = if_else(variable == "pop_d_scaled", mode/summary_table$pop_d_sd, mode),

)

fixed_effect1 <- fixed_effect1 %>%

mutate(mean = if_else(variable == "poor_scaled", mean/summary_table$poor_sd, mean),

sd = if_else(variable == "poor_scaled", sd/summary_table$poor_sd, sd),

lci = if_else(variable == "poor_scaled", lci/summary_table$poor_sd, lci),

uci = if_else(variable == "poor_scaled", uci/summary_table$poor_sd, uci),

mode = if_else(variable == "poor_scaled", mode/summary_table$poor_sd, mode),

)

fixed_effect1 <- fixed_effect1 %>%

mutate(mean = if_else(variable == "hsp_scaled", mean/summary_table$hsp_sd, mean),

sd = if_else(variable == "hsp_scaled", sd/summary_table$hsp_sd, sd),

lci = if_else(variable == "hsp_scaled", lci/summary_table$hsp_sd, lci),

uci = if_else(variable == "hsp_scaled", uci/summary_table$hsp_sd, uci),

mode = if_else(variable == "hsp_scaled", mode/summary_table$hsp_sd, mode),

)

fixed_effect1 <- fixed_effect1 %>%

mutate(mean = if_else(variable == "phc_scaled", mean/summary_table$phc_sd, mean),

sd = if_else(variable == "phc_scaled", sd/summary_table$phc_sd, sd),

lci = if_else(variable == "phc_scaled", lci/summary_table$phc_sd, lci),

uci = if_else(variable == "phc_scaled", uci/summary_table$phc_sd, uci),

mode = if_else(variable == "phc_scaled", mode/summary_table$phc_sd, mode),

)

precision <- result_selected$summary.hyperpar

fitted_values <- result_selected$summary.fitted.values

# model fit stat

model_fit2 <- data.frame(

DIC= result_selected$dic$dic,

WAIC = result_selected$waic$waic

)

model2 <- fixed_effect1 %>%

bind_rows(precision) %>% bind_rows(model_fit2)

write.csv(model2, "model2.csv")

# 3) Spatial CAR Model (Besag Model) with selected covariates (pop_d, poor, hsp)

#merged_data_clean$car_effect <- 1:nrow(merged_data_clean)

merged_data_clean$car_effect <- merged_data_clean$X_id

# Simplified spatial model with only the Besag effect

formula_spatial_test <- DM_more18 ~ 1 + f(car_effect, model = "besag", graph = Q, scale.model = TRUE)

result_spatial_test <- inla(

formula_spatial_test,

data = merged_data_clean,

family = "binomial", # If modeling proportions

Ntrials = merged_data_clean$total_pop, # Total population at risk

control.predictor = list(compute = TRUE),

control.compute = list(dic = TRUE, waic = TRUE)

)

summary(result_spatial_test)

# Check the summary of the results

fixed_effect <- result_spatial_test$summary.fixed

precision <- result_spatial_test$summary.hyperpar

fitted_values <- result_spatial_test$summary.fitted.values

# model fit stat

model_fit3 <- data.frame(

DIC= result_spatial_test$dic$dic,

WAIC = result_spatial_test$waic$waic

)

model3 <- fixed_effect %>% bind_rows(precision) %>% bind_rows(model_fit3)

write.csv(model3, "model3.csv")

# Model 4 Full Spatial CAR model with covariates

formula_spatial <- DM_more18 ~ 1 + pop_d_scaled + poor_scaled + hsp_scaled + phc_scaled + f(car_effect, model = "besag", graph = Q, scale.model = TRUE)

result_spatial <- inla(

formula_spatial,

data = merged_data_clean,

family = "binomial", # If modeling proportions

Ntrials = merged_data_clean$total_pop,

control.predictor = list(compute = TRUE),

control.compute = list(dic = TRUE, waic = TRUE)

)

summary(result_spatial)

fixed_effect <- result_spatial$summary.fixed

fixed_effect1 <- fixed_effect %>% select(-kld) %>%

rownames_to_column(var = "variable") %>%

rename(lci = '0.025quant',

uci = '0.975quant') %>%

mutate(mean = if_else(variable == "pop_d_scaled", mean/summary_table$pop_d_sd, mean),

sd = if_else(variable == "pop_d_scaled", sd/summary_table$pop_d_sd, sd),

lci = if_else(variable == "pop_d_scaled", lci/summary_table$pop_d_sd, lci),

uci = if_else(variable == "pop_d_scaled", uci/summary_table$pop_d_sd, uci),

mode = if_else(variable == "pop_d_scaled", mode/summary_table$pop_d_sd, mode),

)

fixed_effect1 <- fixed_effect1 %>%

mutate(mean = if_else(variable == "poor_scaled", mean/summary_table$poor_sd, mean),

sd = if_else(variable == "poor_scaled", sd/summary_table$poor_sd, sd),

lci = if_else(variable == "poor_scaled", lci/summary_table$poor_sd, lci),

uci = if_else(variable == "poor_scaled", uci/summary_table$poor_sd, uci),

mode = if_else(variable == "poor_scaled", mode/summary_table$poor_sd, mode),

)

fixed_effect1 <- fixed_effect1 %>%

mutate(mean = if_else(variable == "hsp_scaled", mean/summary_table$hsp_sd, mean),

sd = if_else(variable == "hsp_scaled", sd/summary_table$hsp_sd, sd),

lci = if_else(variable == "hsp_scaled", lci/summary_table$hsp_sd, lci),

uci = if_else(variable == "hsp_scaled", uci/summary_table$hsp_sd, uci),

mode = if_else(variable == "hsp_scaled", mode/summary_table$hsp_sd, mode),

)

fixed_effect1 <- fixed_effect1 %>%

mutate(mean = if_else(variable == "phc_scaled", mean/summary_table$phc_sd, mean),

sd = if_else(variable == "phc_scaled", sd/summary_table$phc_sd, sd),

lci = if_else(variable == "phc_scaled", lci/summary_table$phc_sd, lci),

uci = if_else(variable == "phc_scaled", uci/summary_table$phc_sd, uci),

mode = if_else(variable == "phc_scaled", mode/summary_table$phc_sd, mode),

)

precision <- result_spatial$summary.hyperpar

fitted_values <- result_spatial$summary.fitted.values

# model fit stat

model_fit4 <- data.frame(

DIC= result_spatial$dic$dic,

WAIC = result_selected$waic$waic

)

model4 <- fixed_effect1 %>%

bind_rows(precision) %>% bind_rows(model_fit4)

write.csv(model4, "model4.csv")

#Model 5: BYM2 model (spatial + unstructured random effects)

formula_bym2 <- DM_more18 ~ 1 + f(re_u, model = "bym2", graph = Q, scale.model = TRUE)

result_bym2 <- inla(

formula_bym2,

data = merged_data_clean,

family = "binomial",

Ntrials = merged_data_clean$total_pop,

control.predictor = list(compute = TRUE),

control.compute = list(dic = TRUE, waic = TRUE)

)

summary(result_bym2)

# Check the summary of the results

fixed_effect <- result_bym2$summary.fixed

precision <- result_bym2$summary.hyperpar

fitted_values <- result_bym2$summary.fitted.values

# model fit stat

model_fit5 <- data.frame(

DIC= result_bym2$dic$dic,

WAIC = result_bym2$waic$waic

)

model5 <- fixed_effect %>%

bind_rows(precision) %>% bind_rows(model_fit5)

write.csv(model5, "model5.csv")

# Model 6: BYM + Spatial + Unstructured Random Effect Model (BYM2) using re_u

merged_data_clean$re_u <- merged_data_clean$X_id

formula_bym <- DM_more18 ~ 1 + pop_d_scaled + poor_scaled + hsp_scaled + phc_scaled + f(re_u, model = "bym2", graph = Q, scale.model = TRUE)

# Fit the BYM2 model

result_BymFinal <- inla(

formula_bym,

data = merged_data_clean,

family = "binomial",

Ntrials = merged_data_clean$total_pop,

control.predictor = list(compute = TRUE),

control.compute = list(dic = TRUE, waic = TRUE, config = TRUE)

)

# Summary of the results

summary(result_BymFinal)

# Check the summary of the results

fixed_effect <- result_BymFinal$summary.fixed

fixed_effect1 <- fixed_effect %>%

rownames_to_column(var = "variable") %>%

rename(lci = '0.025quant',

uci = '0.975quant') %>%

mutate(mean = if_else(variable == "pop_d_scaled", mean/summary_table$pop_d_sd, mean),

sd = if_else(variable == "pop_d_scaled", sd/summary_table$pop_d_sd, sd),

lci = if_else(variable == "pop_d_scaled", lci/summary_table$pop_d_sd, lci),

uci = if_else(variable == "pop_d_scaled", uci/summary_table$pop_d_sd, uci),

mode = if_else(variable == "pop_d_scaled", mode/summary_table$pop_d_sd, mode),

)

fixed_effect1 <- fixed_effect1 %>%

mutate(mean = if_else(variable == "poor_scaled", mean/summary_table$poor_sd, mean),

sd = if_else(variable == "poor_scaled", sd/summary_table$poor_sd, sd),

lci = if_else(variable == "poor_scaled", lci/summary_table$poor_sd, lci),

uci = if_else(variable == "poor_scaled", uci/summary_table$poor_sd, uci),

mode = if_else(variable == "poor_scaled", mode/summary_table$poor_sd, mode),

)

fixed_effect1 <- fixed_effect1 %>%

mutate(mean = if_else(variable == "hsp_scaled", mean/summary_table$hsp_sd, mean),

sd = if_else(variable == "hsp_scaled", sd/summary_table$hsp_sd, sd),

lci = if_else(variable == "hsp_scaled", lci/summary_table$hsp_sd, lci),

uci = if_else(variable == "hsp_scaled", uci/summary_table$hsp_sd, uci),

mode = if_else(variable == "hsp_scaled", mode/summary_table$hsp_sd, mode),

)

fixed_effect1 <- fixed_effect1 %>%

mutate(mean = if_else(variable == "phc_scaled", mean/summary_table$phc_sd, mean),

sd = if_else(variable == "phc_scaled", sd/summary_table$phc_sd, sd),

lci = if_else(variable == "phc_scaled", lci/summary_table$phc_sd, lci),

uci = if_else(variable == "phc_scaled", uci/summary_table$phc_sd, uci),

mode = if_else(variable == "phc_scaled", mode/summary_table$phc_sd, mode),

)

precision <- result_BymFinal$summary.hyperpar

fitted_values <- result_BymFinal$summary.fitted.values

# model fit stat

model_fit6 <- data.frame(

DIC= result_BymFinal$dic$dic,

WAIC = result_BymFinal$waic$waic

)

model6 <- fixed_effect1 %>%

bind_rows(precision) %>% bind_rows(model_fit6)

write.csv(model6, "model6.csv")

#Coefficients and CrI

# Extract fixed effects (mean, standard deviation, quantiles)

fixed_effects <- result_BymFinal$summary.fixed

print(fixed_effects)

######################

#########################################

# Extract fitted values (posterior means)

fitted_values_bym <- result_BymFinal$summary.fitted.values$mean

# Add fitted values to the merged_data_clean

merged_data_clean <- merged_data_clean %>%

mutate(fitted_values_bym_dm_q = fitted_values_bym)

# Create the new variable DM_Prop

merged_data_clean <- merged_data_clean %>%

mutate(DM_Prop = DM_more18 / total_pop)

# Add quantiles to the data (0.025 and 0.975 quantiles)

merged_data_clean <- merged_data_clean %>%

mutate(

lower_quantile_bym_dm_q = result_BymFinal$summary.fitted.values[, "0.025quant"],

upper_quantile_bym_dm_q = result_BymFinal$summary.fitted.values[, "0.975quant"]

)

# Extract the spatial (structured) random effects

# Ensure you only extract the spatial random effects that match the number of regions

spatial_effects_bym <- result_BymFinal$summary.random$re_u$mean[1:nrow(merged_data_clean)]

# Add the spatial random effects to merged_data_clean

merged_data_clean <- merged_data_clean %>%

mutate(spatial_effects_bym_dm_q = spatial_effects_bym)

# Extract fitted values (posterior means)

fitted_values <- result_BymFinal$summary.fitted.values$mean

# Calculate exceedance probabilities: P(prevalence > 0.025)

threshold <- 0.025

exceedance_prob <- 1 - pbinom(threshold * merged_data_clean$total_pop,

size = merged_data_clean$total_pop,

prob = fitted_values)

# Add exceedance probabilities to the spatial object

merged_data_clean$exceedance_prob_dm_q <- exceedance_prob

final_data_clean <- merged_data_clean %>%

select(c(X_id:District), c(fitted_values_bym_dm_q:exceedance_prob_dm_q))

write.csv(final_data_clean, "DM_Q_Result.csv", row.names = FALSE)

# Joint exceedance probability

# Use national prevalence thresholds

q_TB <- 0.0028

q_DM <- 0.025

# Extract fitted values

fitted_TB <- result_BymFinaltb$summary.fitted.values$mean

fitted_DM <- result_BymFinal$summary.fitted.values$mean

# Draw posterior samples

samples_TB <- inla.posterior.sample(1000, result_BymFinaltb)

samples_DM <- inla.posterior.sample(1000, result_BymFinal)

# Function to extract fitted values from latent field

extract_fitted <- function(samples, index) {

sapply(samples, function(x) {

eta <- x$latent[index] # linear predictor

p <- exp(eta) / (1 + exp(eta)) # inverse logit to get prevalence

return(p)

})

}

# Number of districts

n_districts <- length(result_BymFinaltb$summary.fitted.values$mean)

# Initialize vector

joint_exceedance <- numeric(n_districts)

# Loop through each district

for (i in seq_len(n_districts)) {

TB_vals <- extract_fitted(samples_TB, i)

DM_vals <- extract_fitted(samples_DM, i)

# Proportion of samples where both exceed thresholds

joint_exceedance[i] <- mean(TB_vals > q_TB & DM_vals > q_DM)

}

# Add joint exceedance to spatial data

merged_data_cleantb$joint_exceedance <- joint_exceedance

# Data validation and sensitivity analysis

# Define number of folds

K <- 5

# Assign each area to a fold (randomly for now; spatial balance requires clustering or stratification)

set.seed(123)

merged_data_clean$fold <- sample(1:K, nrow(merged_data_clean), replace = TRUE)

# Create empty prediction column

merged_data_clean$predicted <- NA

for (k in 1:K) {

train_data <- merged_data_clean %>% filter(fold != k)

test_data <- merged_data_clean %>% filter(fold == k)

full_data <- bind_rows(train_data, test_data)

result_full <- inla(

formula_bym,

data = full_data,

family = "binomial",

Ntrials = full_data$total_pop,

control.predictor = list(compute = TRUE),

control.compute = list(dic = TRUE, waic = TRUE)

)

test_ids <- test_data$X_id

merged_data_clean$predicted[merged_data_clean$X_id %in% test_ids] <- result_full$summary.fitted.values$mean[full_data$X_id %in% test_ids]

}

# Check for missing predictions

table(is.na(merged_data_clean$predicted))

# Compute RMSE

rmse <- sqrt(mean((merged_data_clean$DM_more18 / merged_data_clean$total_pop - merged_data_clean$predicted)^2, na.rm = TRUE))

print(paste("RMSE:", round(rmse, 4)))

as.data.frame(rmse) %>%

export(here("Rmse_dm_dist.csv"))

#PIT Hisoram

# PIT histogram using fitted values

pit_values <- pnorm(merged_data_clean$DM_more18 / merged_data_clean$total_pop,

mean = merged_data_clean$predicted,

sd = sqrt(merged_data_clean$predicted * (1 - merged_data_clean$predicted) / merged_data_clean$total_pop))

# Create the plot

pit_plot <- ggplot(data.frame(pit = pit_values), aes(x = pit)) +

geom_histogram(bins = 20, fill = "skyblue", color = "black") +

labs(

title = "PIT Histogram",

x = "PIT Value",

y = "Frequency"

) +

theme_minimal(base_size = 14) +

theme(

plot.title = element_text(hjust = 0.5, face = "bold"),

axis.title = element_text(face = "bold")

)

# Save as TIFF

tiff("DM_dist_PIT_Histogram_prior.tiff", width = 6, height = 4, units = "in", res = 300, compression = "lzw")

# Weighted BYM2 model

result_BymFinal <- inla(

formula_bym,

data = merged_data_clean,

family = "binomial",

Ntrials = merged_data_clean$total_pop,

weights = merged_data_clean$weight_dm18,

control.predictor = list(compute = TRUE),

control.compute = list(dic = TRUE, waic = TRUE)

)

# Distance-based analysis

# Project to a Metric CRS

shp <- st_transform(indonesia, 23830)

# calcualate centroid to estimate distance

centroid <- st_centroid(shp)

# Get centroids of each region for spatial modelling

coords <- st_coordinates(st_centroid(shp))

# Define neighbors within a threshold distance (e.g., 100 km)

nb <- dnearneigh(coords, 0, 100000) # distance in meters

# BYM + Spatial + Unstructured Random Effect Model (BYM2) using re_u

merged_data_clean$re_u <- merged_data_clean$X_id

formula_bym <- DM_more18 ~ 1 + pop_d_scaled + poor_scaled + hsp_scaled + phc_scaled + f(re_u, model = "bym2", graph = Q)

# Fit the BYM2 model

result_BymFinal <- inla(

formula_bym,

data = merged_data_clean,

family = "binomial",

Ntrials = merged_data_clean$total_pop,

weights = merged_data_clean$weight_dm18,

control.predictor = list(compute = TRUE),

control.compute = list(dic = TRUE, waic = TRUE)

)
